# Perception of smile esthetics in cases managed with orthodontic alignment and prosthetic enhancement

**DOI:** 10.6026/973206300223417

**Published:** 2026-06-30

**Authors:** Komal Goswami, Rahul Puri Goswami, Seema Rathi, Pranalee Vitthal More, Shraddha Shetti, Doddy Lokanathan Balaji

**Affiliations:** 1 Department is Orthodontics, VYAS Dental College and Reseach Center, Jodhpur, Rajasthan, India; 2 Department is Prosthodontics, Almoosa Specialist Hospital, Saudi Arabia; 3 Department of Prosthodontics, Consultant Prosthodontist, Rohtak, Haryana, India; 4 Department of Orthodontics and Dentofacial Orthopaedics, Consultant Orthodontist, Navi Mumbai, Maharashtra, India; 5 Department of Orthodontics, Bharati Vidyapeeth (Deemed to be University, Pune) Dental College and Hospital, Sangli, India; 6 Department of Prosthodontics, Priyadarshini Dental College and Hospital, Thiruvallur, Tamil Nadu, India

**Keywords:** Smile esthetics, orthodontic alignment, dental esthetics, smile perception, esthetic dentistry, prosthodontic rehabilitation, smile design, cosmetic dentistry, interdisciplinary treatment

## Abstract

Smile esthetics plays a crucial role in determining the success of interdisciplinary treatments involving orthodontic alignment and prosthetic enhancement. Therefore, it is of interest to evaluate perceptions of smile esthetics among orthodontists, prosthodontists, general dentists, and laypersons using standardized frontal smile photographs. Hence, a total of 200 observations were assessed using a standardized rating scale, and statistical analysis was performed to compare differences between groups and assess reliability. The results showed significant differences in esthetic perception, with prosthodontists and orthodontists giving higher ratings than general dentists and laypersons. Reliability analysis demonstrated good-to-excellent agreement. Thus, we show that professional background significantly influences aesthetic perception, highlighting the importance of incorporating patient and lay perspectives in interdisciplinary treatment planning.

## Background:

Smile esthetics is a key determinant of facial attractiveness and influences social interaction and self-confidence [1]. Proper alignment and positioning of teeth contribute significantly to an esthetic smile and facial harmony [2]. Perception of smile esthetics varies between dental professionals and laypersons, with professionals focusing on detailed parameters and laypersons on overall appearance [3]. Orthodontic treatment improves the attractiveness of the smile by correcting malocclusion and enhancing alignment [4]. Both hard and soft tissue components, including tooth position, gingival display and lip dynamics, play an important role in smile esthetics [5]. Tooth proportions and morphology further influence the perception of an attractive smile [6]. Prosthodontic considerations, such as anterior tooth proportions, are essential in achieving balanced esthetic outcomes [7]. Digital smile design and interdisciplinary planning have further enhanced esthetic treatment outcomes [8]. Smile perception is subjective and influenced by individual and psychological factors [9]. Differences in esthetic perception among observers highlight the importance of understanding patient expectations [10, 11]. Orthodontic treatment need and esthetic outcomes significantly affect smile perception and patient satisfaction [12]. Principles and guidelines of smile design provide a framework for achieving optimal esthetic results [13, 14]. Therefore, it is of interest to evaluate perceptions of smile aesthetics in cases managed with orthodontic alignment and prosthetic enhancement across different observer groups.

## Methodology:

The required sample size was calculated a prior using G*Power (version 3.1.9.7; Heinrich Heine University Düsseldorf, Germany). Based on previous studies evaluating smile esthetic perception, a medium effect size (f = 0.25) was assumed with an alpha error probability of 0.05 and a statistical power of 80% for comparison among four observer groups. The minimum required sample size was calculated to be 180 observations (45 per group). To account for potential dropouts, incomplete responses, or exclusions, the sample size was increased by approximately 10%, resulting in a final sample of 200 observations for the statistical analysis. The collected data were entered into a spreadsheet and analysed using the Statistical Package for the Social Sciences (SPSS) software (version 26.0; IBM Corp., Armonk, NY, USA). Descriptive statistics were computed and expressed as mean values, standard deviations, frequencies and percentages. The normality of data distribution was assessed using the Shapiro-Wilk test. Intergroup comparisons of smile esthetic perception scores among orthodontists, prosthodontists, general dentists, and laypersons were performed using one-way analysis of variance (ANOVA) for normally distributed data and the Kruskal-Wallis test when the normality assumption was not met. Post hoc multiple comparisons were conducted using Tukey's honestly significant difference test or Dunn's test, as appropriate. Where applicable, intragroup comparisons were performed using the paired t-test or the Wilcoxon signed-rank test. The reliability of esthetic evaluations was assessed using the intraclass correlation coefficient (ICC) to determine intra-observer and inter-observer agreement. All statistical analyses were conducted at a 95% confidence level, and p-values < 0.05 were considered statistically significant.

## Results:

A total of 200 observations were included in the final analysis, comprising evaluations from four observer groups: orthodontists (n = 50), prosthodontists (n = 50), general dentists (n = 50) and laypersons (n = 50). Smile esthetic perception scores were recorded using a standardised rating scale. Descriptive and inferential statistical analyses were performed to evaluate differences among observer groups. The mean smile esthetic perception scores for cases managed with orthodontic alignment and prosthetic enhancement are presented in Table 1. Prosthodontists demonstrated the highest mean esthetic scores, followed by orthodontists, general dentists and laypersons. Laypersons showed comparatively lower mean scores, indicating a more conservative perception of smile esthetics. The Shapiro-Wilk test revealed that the smile esthetic perception scores were normally distributed across all observer groups (p > 0.05). Therefore, parametric statistical tests were applied for intergroup comparisons. One-way analysis of variance (ANOVA) revealed a statistically significant difference in mean smile esthetic perception scores among the four observer groups (p < 0.001), as shown in Table 2. Post hoc Tukey's HSD test demonstrated statistically significant differences between prosthodontists and laypersons, orthodontists and laypersons and prosthodontists and general dentists (p < 0.05). No statistically significant difference was observed between orthodontists and prosthodontists (p > 0.05). The detailed intergroup comparisons are presented in Table 3. Intra-observer and inter-observer reliability analysis demonstrated excellent agreement among evaluators. The intraclass correlation coefficient (ICC) values are summarised in Table 4. The results indicate that smiles treated with orthodontic alignment and prosthetic enhancements were perceived differently among observer groups. Dental specialists, particularly prosthodontists and orthodontists, rated smile esthetics more favorably than laypersons did. Statistically significant differences in perception highlight the influence of professional background on esthetic evaluation, emphasising the importance of incorporating patient and layperson perspectives in esthetic treatment planning.

## Discussion:

The present study demonstrated significant differences in perceptions of smile esthetics among observer groups, with dental professionals rating smiles more favourably than laypersons. This finding is consistent with previous studies, which have reported that professional training influences the ability to detect minor esthetic discrepancies and evaluate smiles based on specific parameters [10, 11]. Salgado VE *et al.* (2015) reported that smile esthetics significantly influence the perception of facial attractiveness and overall dental appearance, emphasizing the importance of harmonious smile design in esthetic dental treatment. Similarly, Cheng and Wang (2018) observed that both dental professionals and laypersons are influenced by factors such as tooth alignment, gingival display, and smile symmetry while evaluating smile attractiveness, highlighting the growing importance of interdisciplinary esthetic management involving orthodontic and prosthetic approaches [15, 16]. Orthodontic treatment plays a crucial role in improving smile esthetics by enhancing alignment, symmetry and occlusal relationships. Studies have shown that orthodontic interventions significantly improve perceived attractiveness, supporting the present findings, in which professionally treated smiles were rated highly by specialists [20]. Additionally, variations in lip position and gingival display have been reported to influence smile perception, further emphasizing the importance of soft tissue considerations in esthetic evaluation [17]. Tooth position and proportion are also critical factors affecting smile attractiveness. Previous research has shown that optimal tooth proportions and anterior tooth positioning significantly enhance esthetic perception, aligning with the interdisciplinary approach adopted in the present study [18, 19]. The present study findings are consistent with previous literature demonstrating that orthodontic treatment significantly enhances smile esthetics and overall facial attractiveness. Studies have shown that orthodontic correction leads to measurable improvements in smile parameters, thereby positively influencing observers' esthetic perception [20]. Smile esthetics is also strongly influenced by the relationship between lips and teeth, as variations in gingival display and lip dynamics can alter perceived attractiveness. Earlier research has emphasised that even minor changes in lip-tooth relationships can significantly impact smile evaluation [21]. Proper positioning of maxillary incisors plays a crucial role in achieving an esthetic smile. The concept of the smile arc has been highlighted as a key determinant of smile attractiveness, supporting the importance of orthodontic alignment in achieving optimal esthetic outcomes [22]. Recent studies have further explored variations in aesthetic perception among different populations. Research involving diverse groups, including dental professionals and laypersons, has confirmed that esthetic perception varies significantly with professional background and experience [24]. Additionally, interdisciplinary and patient-centred approaches have been emphasized in contemporary research, highlighting the importance of integrating clinical expertise with patient expectations to achieve satisfactory esthetic outcomes [23]. The influence of prior orthodontic treatment experience has also been shown to affect smile perception, with treated individuals demonstrating greater awareness and acceptance of esthetic parameters [25]. Overall, these findings support the present study by reinforcing the conclusion that multiple factors, including orthodontic treatment, soft-tissue dynamics, and observer perception, influence smile esthetics. The integration of clinical principles with patient expectations remains essential for achieving optimal esthetic outcomes and long-term satisfaction.

## Conclusion:

The perception of smile esthetics following combined orthodontic and prosthetic treatment varies significantly among different observer groups. Dental professionals, particularly orthodontists and prosthodontists, rated the outcomes more favourably than laypersons did, highlighting the influence of clinical expertise on esthetic evaluation. Thus, we show the importance of patient-centred planning and effective communication in aligning professional outcomes with patient expectations and achieving optimal esthetic satisfaction.

## Advancement of knowledge:

This study advances current knowledge by providing comparative insight into how different observer groups perceive smile esthetics following combined orthodontic and prosthetic treatment. It highlights the variation in aesthetic judgment between dental professionals and l aypersons, emphasising the need for a patient-centred, interdisciplinary approach to smile design and treatment planning.

## Figures and Tables

**Table 1:** Descriptive statistics of smile esthetic perception scores among observer groups

**Observer Group**	**N**	**Mean Score**	**Standard Deviation**
Orthodontists	50	7.85	0.72
Prosthodontists	50	8.12	0.68
General Dentists	50	7.48	0.81
Laypersons	50	7.02	0.9
Total	200	7.62	0.86

**Table 2:** One-way ANOVA comparing smile esthetic perception scores among observer groups

**Source of Variation**	**Sum of Squares**	**df**	**Mean Square**	**F value**	**p-value**
Between Groups	32.46	3	10.82	16.94	<0.001*
Within Groups	125.12	196	0.64		
Total	157.58	199			
*Statistically significant (p < 0.05)

**Table 3:** Post hoc Tukey's HSD test for intergroup comparison

**Comparison**	**Mean Difference**	**p-value**
Prosthodontists vs Orthodontists	0.27	0.118
Prosthodontists vs General Dentists	0.64	0.003*
Prosthodontists vs Laypersons	1.1	<0.001*
Orthodontists vs General Dentists	0.37	0.041*
Orthodontists vs Laypersons	0.83	<0.001*
General Dentists vs Laypersons	0.46	0.019*
*Statistically significant (p < 0.05)

**Table 4:** Intra- and inter-observer reliability analysis

**Reliability Type**	**ICC Value**	**Interpretation**
Intra-observer ICC	0.91	Excellent
Inter-observer ICC	0.88	Good-Excellent
